# A Medical Report of His Majesty's Colonial Hospital, Hobart Town, Van Dieman's Land, for the Years 1821 and 1822; Showing the Prevailing Diseases of the Colony in the Different Months

**Published:** 1824-06

**Authors:** James Scott

**Affiliations:** R.N.


					t SI9 ]
STATISTICAL MEDICINE.
Abt. I.-
-A Medical Report of His Majesty's Colonial Hospital,
Hobart Town, Van Diemaiis Land, for the Years 1821 and 1S22;
showing the prevailing Diseases of the Colony in the different
Months.
By James Scott, r.n.
Abscessns  12
Acconchements 2
Amenorrhoea ? ? 0
Ambustio  li
Anasarca ????.. 3
Ascites  j
Apoplexia  0
Asthma ?  7
Amaurosis  2
Anorexia ?????. 0
Bubo  1
Catarrhus  11
Cephalalgia 6
Cholera Morbus 3
Chorea St. Vite 0
Colica   <z
Contusio    16
lCynanche Tonsil. 5
lDebilitas   2
Diarrhoea  18
DiseasesofBones 0
Dislocatio
Dysenteria ? ? ? ? 27
Dyspepsia  17
Dentitio difficilis 0
Enteritis   0
Epilepsia ^ 0
F.pistaxis . ...W 0
Erysipelas  1
Eruptio  29
Excoriatio
Febris   8
Fiirunculus ???? 15
Fractura   0
Fistula in Ano ? ? 2
Gastritis   0
Gonorrhoea .... 14
Hydrocephalus 0
Hydrops articuli 0
Haematemesis ? ? 0
Hepatitis   \
Hernia humorali> 3
Hernia  5
Haeniorrhois ....
Hematocele- ? ?. 0
Hyd 10thorax .. 0
232
15 16
6
0 0
11 9
2 1
1 0
0 0
6
0 0
0, 0
0 0
5 2
5
0 0
0 1
0 3
21 18
1 2
2 1
16 8
0 1
1 1
54 28
25 1?
0 O
0 0
1 0
0 1
5 2
22
1 5
9 7
13 7
1 1
2 1
0 1
9 12
0 0
0 0
0 0
3 6
1 1
6 7
3 2
0 0
0 0
277 247,204 188
23
4
0
15
0
0
1
1
0
0
1
4
0
0
0
2
25
2
2
15
0
0
37
11
1
0
0
0
1
32
1
8
6
3
0
0
7
0
0
0
4
1
7
3
0
1
218
12 7 9
111
0 0 0
9 10 5
1 0 3
10 0
Oil
15 3
0 0 0
0 0 0
0 3
4 4 4
0 4 10
0 0 0
0 0 0
3 1 1
31) 21 29
3 6 0
0 10
15 29 24
1 1 1
0 0 0
30 16 7
4 4 2
000
000
0 1 1
1 0 0
2 <4. 1
32 26 35
2 4 1
11 5 9
10 1() 8
0 1 1
0 0 0
0 0 0
12 9 10
0 0 0
0 0 0
0 0 0
4 13
0 12
7 6 6
8 5 4
0 0
0 1
204 l86'lfi4 238 228
3 8 151
0 2 26
1 0 S
7 7 109
3 n 15
0 0 4
0
0 3
0 33
0 3
1 29
0 10
9 68
15 73
0 4
0 1
6 22
29 332
5 36
0 12
17 218
0 9
0. 11
16 333
1 123
0 2
0 2
1 19
4 11
0 25
25 312
4 39
8 100
12 115
2 16
0 10
0 1
12 121
1 1
0 1
0 1
6 41
3 17
8 77
2 35
0 1
0 3
204 2608 2469
NO. 304. 3 X
520 Statistical Medicine,
Brought forward
Haemoptysis
Hysteria
Hydrarthus
Icterus
Intnssusceptio
Insanitas
Leucorrliea ????
Leus Venerea, )
secondary cases J
Menorrhagia ? ?
Ophthalmia ????
Obstipatio ????
Odontalgia
Otitis
Palpitatio Cordis
Paronychia ? ?
Paracusis ????
Paraphymosis
Phymosis
Phlegmon
Phthisis Pulmon.
Paralysis
Pneumonia ????
Plethora ? ? ? ?
Prolapsus Ani ? ?
Pernio
Rheumatismus ? ?
Scorbutus
Scrofula
Subluxatio ????
Striotura
Tumores
Tetanus
Tinea Capitis ? ?
Ulcus
Urricaria
Vavix
Vermin
Vernca
Vulnus
Vermes
Vaccinatio
Vertigo
Urinary complats,
232
1
0
1
2
0
0
0
277
1
0
0
7
0
0
0
247
1
1
1
0
0
0
0
204
1
0
1
1
0
0
188
1
0
0
0
1
i
0
218 204
0 1
0 o
0 0
0 0
o| o
0
0
0 0
9 13
19 18
5 7
0 0
0 1
5 3
1 0
1 0
0 0
2
1 0
2 3
25 28
bp
<
186
1
0
0
0
0
1
0
184 238 228 204 2608
1 ? 3 15
1 2 1 5
1 9
1 1 2 14
0 0 0 1
0 0 0
Oil
445 475 465 434 415 426 402,442 4l0 451 424 427 5216 5137 57 22
23
0 0 0 1
7 17 15 149
22 19 37 295
16 13 12 104
0 0 1 5
0 0 0 1
3 1 2 24
0 0 0
10 0
0()0 1
2 4 27
0 0 0 7
10 0
14 25 17 279
0 0 0
0 2 0 6
0 0 0 1
37 32 37 437
2 l 3 23
13 07
0
0
47
8
OJ 0
0 0
11
1 1 0
0 0 1
0 0
0 0
26 29
1 0
0 0
S
75
26
15
3
1
461
10
3
433
32
43
2
18
Remarks.
In making a general and brief remark on the accompanying Report, T
shall subjoin a list of diseases which, as yet, have not been observed in
the colony; and, respecting the medical topography of the country, I
have to observe, that i|. is not less peculiar in this respect than it is.in its
vegetable and animal productions, as situations are here enjoyed with
health aud pleasure, which any where else would be considered as in-
Medical and Physical Intelligence. 521
levitable destruction, or hazardous to human life. And, although the
vicissitudes of the thermometer from heat to cold, and of the barometer
from clear weather to foul, are frequent and sudden, they are not suc-
ceeded by the same baneful consequences to the human body as in other
countries: nor are these changes followed by epidemic or contagious
diseases, which as yet can scarcely be said to have appeared. Vaccine
virus has been introduced from the Isle of France and Sydney, buf,
after passing through one or two patients, has become ineffective ; and
I strongly suspect that the same observation is good respecting the
primary symptoms cf the venereal disease, as I have not met with any
cases of it, but in personsPtfifect'from Sydney or the Isle of France.
The diseases, both acute and chronic, are generally mild and of short
duration, and yield more easily to the usual remedies than in any other
country with which I am acquainted. It is to be observed, that a
number of the cases inserted in the foregoing Report were brought on
by intemperance, partial clothing, and exposure to wet and cold; and
that the greater part were European prisoners, of dissolute habits and
broken constitutions, predisposed to disease ; but, such is the salubrity
of the climate to Europeans, that the stamina of their constitutions,
after a short residence, becomes renovated and invigorated, enabling
many to become useful members of society, who were never so before.
In short, the valetudinarian, searching for health, will no where find a
climate and country more congenial to his wishes than Van Diemau's
Land.
A list of Diseases which have not been met with in Van Dicman's
Land.?Intermittent fever, malignant sore-throat, small-pox, measles,
Scarlet fever, hooping-congb, cancer, hydrophobia, diabetes, syphilis,
&c. &c.
H. M. Col. Hospital, Hobart Town ;
1st October, 1823.

				

